# Modularity of Protein Folds as a Tool for Template-Free Modeling of Structures

**DOI:** 10.1371/journal.pcbi.1004419

**Published:** 2015-08-07

**Authors:** Brinda Vallat, Carlos Madrid-Aliste, Andras Fiser

**Affiliations:** Department of Systems and Computational Biology, Albert Einstein College of Medicine, Bronx, New York, New York, United States of America; CNAG - Centre Nacional d’Anàlisi Genòmica and CRG - Centre de Regulació Genòmica, SPAIN

## Abstract

Predicting the three-dimensional structure of proteins from their amino acid sequences remains a challenging problem in molecular biology. While the current structural coverage of proteins is almost exclusively provided by template-based techniques, the modeling of the rest of the protein sequences increasingly require template-free methods. However, template-free modeling methods are much less reliable and are usually applicable for smaller proteins, leaving much space for improvement. We present here a novel computational method that uses a library of supersecondary structure fragments, known as Smotifs, to model protein structures. The library of Smotifs has saturated over time, providing a theoretical foundation for efficient modeling. The method relies on weak sequence signals from remotely related protein structures to create a library of Smotif fragments specific to the target protein sequence. This Smotif library is exploited in a fragment assembly protocol to sample decoys, which are assessed by a composite scoring function. Since the Smotif fragments are larger in size compared to the ones used in other fragment-based methods, the proposed modeling algorithm, SmotifTF, can employ an exhaustive sampling during decoy assembly. SmotifTF successfully predicts the overall fold of the target proteins in about 50% of the test cases and performs competitively when compared to other state of the art prediction methods, especially when sequence signal to remote homologs is diminishing. Smotif-based modeling is complementary to current prediction methods and provides a promising direction in addressing the structure prediction problem, especially when targeting larger proteins for modeling.

## Author Summary

Each protein folds into a unique three-dimensional structure that enables it to carry out its biological function. Knowledge of the atomic details of protein structures is therefore a key to understanding their function. Advances in high throughput experimental technologies have lead to an exponential increase in the availability of known protein sequences. Although strong progress has been made in experimental protein structure determination, it remains a fact that more than 99% of structural information is provided by computational modeling methods. We describe here a novel structure prediction method, SmotifTF, which uses a unique library of known protein fragments to assemble the three-dimensional structure of a sequence. The fragment library has saturated over time and therefore provides a complete set of building blocks required for model building. The method performs competitively compared to existing methods of structure prediction.

## Introduction

The revolution in DNA sequencing technologies over the last decade has resulted in an enormous, and ever growing, number of gene sequences, which is doubling every ~18 months [1–3]. At the same time, the number of experimentally determined protein structures has increasingly lagged behind due to the inherently slower, more expensive and less predictable outcomes of these experiments [4]. The size of the sequence databases has increased 100 fold between 2000 and 2015, reaching 60 million entries. At the same time, the rate of protein structure determination has been much slower, with only ~110,000 total entries in the Protein Data Bank (PDB) [5]. Over the past decade, all structural biology efforts, including structural genomics [6], have led to an overall increase in the structural coverage of existing proteins from ~30% to 40% at the residue level, despite the huge growth of the underlying sequence database. With existing technologies and strategies, it is projected [7] that it would take 15 years to reach a level of ~55% coverage, which was shown to provide considerable utility for defining large-scale functional characterization of organism-specific properties (e.g., the full metabolic network in *Thermotoga maritima* [8]). However, these efforts are now predicted to take twice as long in the expected absence of the Structural Genomics (SG) efforts, as SG centers contributed 50–60% of novel coverage despite accounting for less than 10% of all structure depositions [7]. Therefore, the need for reliable methods to model protein structures is stronger than ever before.

Computational protein structure prediction can be broadly classified into two categories: (a) Homology modeling or template-based modeling (TBM) has been successfully used for modeling protein sequences that have an overall detectable sequence similarity over their entire sequence with an experimentally determined protein structure [9]. (b) *Ab initio* or template-free modeling (TFM) [10] is required for those proteins that do not have any statistically significant similar protein sequences with known structures. Hence, structure prediction has to be carried out using alternative approaches such as fragment assembly [11–14] or using first principles from physics-based methods [15–17]. Homology modeling approaches have limited applicability, but provide more accurate models when compared to template-free prediction methods and has no size limitations [9, 18]. Alternately, there are also hybrid modeling methods, which use indirect experimental information, often obtained from automated or semi-automated high throughput experiments, to provide limited structural restraints that can be used for modeling [19, 20].

Currently, the prospect of increasing structural coverage is tied to the applicability of homology modeling, which provides more than 99.5% of the currently observed ~40% structural coverage of protein sequences [7]. Conservation of protein structure is much higher than that of sequence [21, 22], which results in a comparatively small number of distinct structural families [23]. The size distribution of protein fold families is very uneven and the most frequently occurring folds (e.g., Immunoglobulin, TIM barrel, Rossman fold) have likely already been identified [24, 25]. In a typical genome the 10 most populous superfolds cover a third of the protein sequences [26]. Therefore, homology modeling can provide structural models for thousands of proteins in a typical genome using only a few dozen popular folds as templates, and it is currently almost the single source for three-dimensional models [27, 28]. However, the usefulness of homology modeling is exponentially decreasing as smaller and smaller protein families or singletons need to be modeled. These latter proteins either require a targeted experimental exploration, which is often cost prohibitive, or must be modeled by *ab initio* or “template-free” style approaches, which do not depend on a detectable sequence similarity to a known experimental structure. However, these approaches are currently suitable to model only relatively small proteins and have a limited success rate [18] leaving much room for improvement. Recent Critical Assessment of Techniques for Structure Prediction (CASP) experiments [18, 29] have also reinforced the fact that efforts need to be concentrated on developing template-free prediction methods that can model structures of proteins with little or no information from other protein structures.

Template-free prediction methods can further be classified into two categories: (1) *Ab initio* prediction is the modeling of protein structures from first principles without using any information from other existing protein structures [15–17]. (2) Fragment assembly based methods use a library of protein fragments obtained from known protein structures [11–14] to explore the structure space accessible to the query protein. The fragments themselves may be obtained from remote homologs that share very weak sequence similarity with the query protein and are typically not good enough to be used directly for homology modeling. While the physics-based methods have progressed over the years, they have been less successful than the fragment assembly based methods [10]. The various fragment assembly approaches differ mainly in the type of fragments and the energy functions used for sampling and scoring decoys. I-tasser [12] and Tasser [14] use fragments of aligned segments in varying sizes obtained from various threading methods and follow a replica-exchange Monte Carlo sampling for generating full models. Another variant of Tasser is chunk-Tasser [30], which uses fragments of supersecondary structures consisting of three consecutive secondary structures as fragments and folds them independently to obtain restrains for modeling. Rosetta [11] uses a library of three and nine residue fragments obtained from remote homologs identified from Psi-BLAST [31] and a simulated annealing Monte Carlo sampling algorithm to obtain protein models. Although most fragment-based methods differ in the type of fragments and the energy functions used for sampling, most of them use a similar approach to score the models. The most commonly used model selection scheme is structural clustering of the sampled decoy structures, to obtain the lowest free energy state, identified as the most populous cluster. Some other methods predict the best model by identifying the consensus conformation from different prediction algorithms [32, 33]. It has been reported that current template-free prediction methods perform best for small single-domain targets with length up to 120 amino acids [10, 18]. The quality of prediction drops significantly for larger proteins since the conformational search becomes tedious and less accurate for larger proteins.

We have developed a fragment assembly based template-free prediction method using a library of supersecondary structure fragments, known as Smotifs. The concept of using a library of protein structure motifs for structure prediction has been explored earlier using a set of locally defined protein motifs known as I-sites (invariant or initiation sites) [34]. I-sites are short sequence motifs of length 3–19 obtained by exhaustive clustering of sequence segments obtained from a non-redundant database of known structures, where each sequence pattern correlates strongly with a recurrent local structural motif in proteins. The I-sites library has been successfully combined with a Hidden Markov Model approach to address various protein sequence and structure related questions such as tertiary and secondary structure prediction, sequence comparison, dihedral angle region prediction and gene identification [35, 36]. A major difference between those studies and the current method is the definition of the motif fragment, which provides a different conceptual context to our structure prediction approach. We define Smotifs as two secondary structure elements in a protein connected by loop. We have created a library of Smotifs from all known protein structures in the PDB [37]. In our earlier study it was observed that the Smotif fragment library is saturated [38] leading to a hypothesis that all known and yet to be discovered protein folds can be generated using different combinations of the Smotifs already present in the library [39]. Subsequently, the Smotif library was successfully used to develop a hybrid modeling method using chemical shift data from NMR experiments [19], to classify [40] and model loops in protein structures [37, 41] and to develop *de novo* structure based design method [42]. Here, we show that the Smotif library can be used to model protein structures using a fragment assembly method, referred to as “SmotifTF”. The new method, SmotifTF is successful in predicting the overall fold for over 50% of the *ab initio* test proteins explored in this study.

## Results

**Fig 1:**
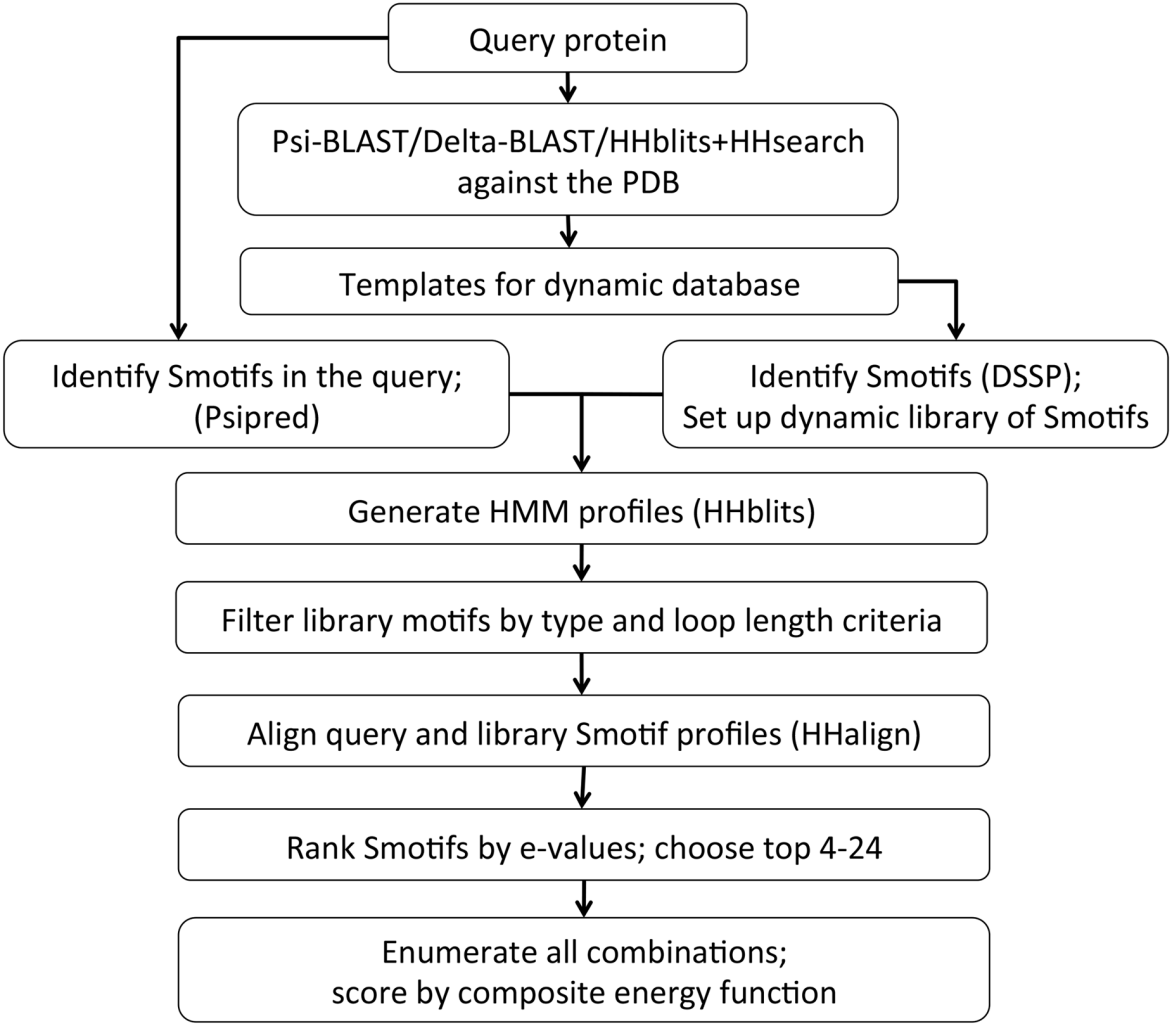
Flowchart of the SmotifTF prediction algorithm.

The modeling algorithm consists of the following steps (Fig 1): First, PSIPRED [43] is used to predict the secondary structures and identify the putative Smotifs in the query protein of interest. Next, suitable Smotif fragments are sampled from a set of related sequences with known three dimensional structures available in the PDB [5]. These sequences are detected using three different methods, Psi-BLAST [31], delta-BLAST [44] and HHblits/HHsearch [45, 46]. From these remote homologs, Smotifs are collected into a “dynamic” Smotif library, tailor-made for the query protein. The Smotif fragments are larger in size (average size is 27.44 amino acid residues per Smotif in the current data set) compared to other existing fragment assembly methods (for instance, Rosetta uses fragments of 3 and 9 residues), making it feasible to carry out an exhaustive enumeration of all possible combinations of the chosen fragments during the decoy sampling step. The scoring function used to select the best model from the decoys is a linear combination of four knowledge-based components: (1) an orientation dependent statistical pair-wise potential using shuffled reference state [47–49], (2) the radius of gyration, (3) main-chain only hydrogen bond potential [50] and (4) an implicit solvation potential [51]. The method is developed on a set of 20 randomly selected proteins that represent different folds and is tested on a set of 16 *ab initio* targets selected from just released PDB entries to avoid any bias. SmotifTF method is compared to other state of the art structure prediction methods, I-tasser [12], Rosetta [11] and HHpred [52], which were chosen because these methods have performed well in recent CASP benchmarking experiments [18, 29]. The results of these predictions are discussed below.

### Performance of the prediction algorithm as a function of the quality of Smotif fragments in the library

**Fig 2:**
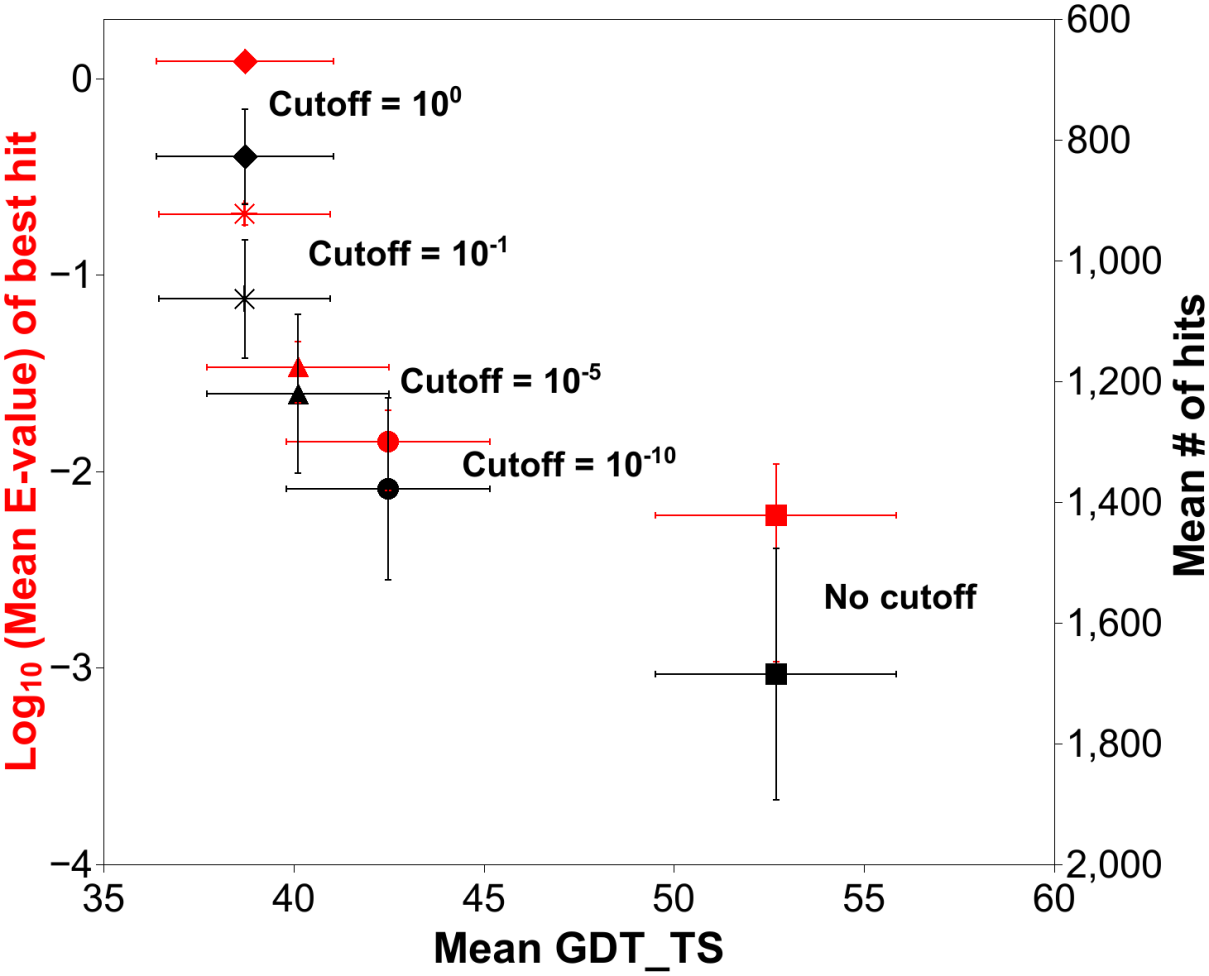
Performance evaluation in the training set. Prediction quality (assessed as the mean GDT_TS of the top-scoring model against the native structure) is plotted on the X-axis for 20 cases at different e-value cutoffs used in generating the dynamic Smotif library. The data points for different e-value cutoffs are shown in different symbols (no cutoff (square), 10^-10^ (circle), 10^-5^ (triangle), 10^-1^ (star) and 10^0^ (diamond)). The dual Y-axes correspond to the mean number of hits in the dynamic Smotif database (right axis, inversed scale, black data points) and to the mean e-value of the best hit in the dynamic database (left axis, log scale, red data points), respectively.

The SmotifTF method was developed on a randomly selected set of 20 proteins. Table 1 summarizes the accuracy of these predictions in terms of the GDT_TS scores [53] of the top-scoring model with regard to the native structure. GDT_TS score calculates the percentage of structurally equivalent pairs of residues at 1, 2, 4 and 8 Å cutoff values upon optimal superposition of the experimentally determined native structure and the computational model. The average GDT_TS score for the predictions in this dataset is 54.67, with 12 protein models above GDT_TS 50% and all proteins with GDT_TS > 30%, indicating correct fold predictions for all proteins. However, this data set is not challenging for template-free predictions, because for many of these cases, good structural templates are available, which make them suitable for homology modeling. To simulate conditions that require template-free structure predictions, the algorithm was repeated by systematically removing high quality templates prior to creating the dynamic Smotif library. All templates with e-values better than 10^-10^, 10^-5^, 10^-1^ and 10^0^ were removed, respectively, and the prediction algorithm was repeated (Table 1). As expected, the quality of prediction depends heavily on the quality of the templates in the Smotif library. The stricter the e-value cutoff for filtering out homologous templates gets, the worse the predictions become (Table 1). The interplay between the quality of prediction (Mean GDT_TS) and the size of the dynamic database (set of Smotifs obtained from remote homologs) at different e-value cutoffs is shown in Fig 2. As the cutoff is made more stringent from “no cutoff” (all possible templates considered) to 10^0^ (all templates with e-value better than 1.0 are excluded), the average number of Smotifs in the dynamic database (right Y-axis) decreases by 50% (from 1684.05 to 826.95) and the average e-value of the best hit in the dynamic database (left Y-axis) increases from barely significant to a random hit value (from 0.006 to 1.225), indicating the gradual loss of reliable templates. The quality of predictions drops from a mean GDT_TS of 54.67 to 38.71, as the e-value cutoffs get stricter from “no cutoffs” to 10^0^, respectively. It has been shown earlier that a practical discriminator between *ab initio* or template-free models and homology models is around GDT_TS 30% and values above GDT_TS 50% indicate high quality homology models [54]. In the current dataset, when a stringent e-value cutoff of 10^0^ is used, 15 of the 20 proteins have GDT_TS > 30%, among which, three have GDT_TS > 50%. All values stay above 20% indicating that the fold, at least partially, has been captured in every case. Even under strict template-free modeling conditions, the SmotifTF prediction method predicts a model above 30% GDT_TS for 75% of the cases, with an overall average GDT_TS of 38.71.

**Table 1:** GDT_TS values of top scoring models obtained with SmotifTF method using dynamic Smotif library generated at different e-value cutoffs.

PDB code and chain	No cutoff	e-value > 10^-10^	e-value > 10^-5^	e-value > 10^-1^	e-value > 10^0^
1aabA	67.06	46.43	46.43	46.43	46.43
1bqzA	58.08	39.62	39.62	39.62	39.62
1dcjA	80.36	45.36	45.36	36.07	35.00
1hdnA	52.44	32.32	32.32	32.32	26.83
1iloA	51.35	47.97	39.87	35.47	27.70
1khmA	40.29	36.46	36.46	36.46	40.28
1lq7A	61.91	61.91	61.91	61.91	61.91
1myoA	63.38	18.20	20.61	20.61	20.61
1ng7A	44.00	44.00	44.00	38.50	38.50
1om2A	45.06	45.60	45.60	45.60	45.06
1pveA	56.13	62.74	62.74	58.96	58.96
1rg6A	37.50	28.33	28.33	32.32	30.00
1uzcA	40.18	38.39	38.39	38.39	38.39
1ss2A	36.72	31.64	30.08	25.78	28.91
1tizA	71.15	55.39	47.69	38.08	41.54
1wgnA	78.13	63.54	49.48	51.04	51.04
1wgwA	62.79	40.99	40.99	40.99	40.00
1wgvA	30.06	27.38	25.60	25.60	25.16
1wjtA	46.43	49.03	37.34	37.34	41.56
1wgqA	70.43	34.14	29.30	31.99	36.29
Mean	54.67	42.47	40.11	38.70	38.71

### Comparison to other prediction methods

Recent CASP experiments show that template-free modeling is still a work in progress and require further methodological developments to be able to provide useful models [29]. Some of the methods that performed the best in the template-free category in recent CASP experiments include I-tasser [12], HHpred [52] and Rosetta [11]. I-tasser and Rosetta are fragment assembly-based methods that use different kinds of fragments and sampling algorithms as described earlier. HHpred is a template-based modeling method, that uses Hidden Markov Model (HMM) profiles and an HMM-HMM comparison algorithm [45] to identify remotely related templates for homology modeling. The HMM-based sequence search is more sensitive and is known to perform better than traditional heuristic sequence search methods.

The benchmarks against the above three methods were carried out on a test set of 16 proteins obtained from weekly new releases of the PDB from 10-08-2015 to 12-31-2015. These were submitted to the I-tasser and HHpred servers online, while Rosetta calculations were carried out using a local installation. In each case, the trivial prediction using the self-template was eliminated. HHpred requires the user to choose the templates for model building, after the HHsearch step. If available, multiple templates were chosen to obtain maximum possible query coverage, which were then submitted to Modeller [55]. In case of Rosetta, 10000 decoys were sampled using the Rosetta algorithm from 100 parallel simulations. The resulting models were then clustered using the algorithm provided in the Rosetta package to identify the largest cluster. The center of the largest cluster was identified as the best model. The results of this analysis are summarized in Table 2. The mean GDT_TS scores show that I-tasser performs the best with a mean GDT_TS score of 36.97, SmotifTF comes in second with an average GDT_TS score of 33.05 and HHpred and Rosetta make the third and fourth positions with GDT_TS 31.56 and 30.70 respectively. The average GDT_TS is comparable in the four methods and is around 30–35%. Each method has some highlight performances, where its prediction is the best compared to the others. For instance, I-tasser has the best prediction for targets 2mpvA, 3wzsA and 4uzxA whereas Rosetta does better with 4nknA and 4rd5A. HHpred has better models for 4ux3B and 4v1am and SmotifTF has better predictions for 4pqzA, 2mpoA, and 4o7kA. In case of 9 of the 16 proteins in this benchmark test set (56%), SmotifTF predicts a model with GDT_TS over 30%, indicating an overall correct fold prediction for the *ab initio* targets. I-tasser, Rosetta and HHpred have predictions above 30% GDT_TS for 9, 9 and 7, respectively. The proteins in the table are sorted based on the e-value of best hit in the PDB (column 4). If one examines the target proteins with the least trivial templates (only high e-value hits are retained in the Smotif library), SmotifTF has an advantage over the other methods as reflected in the mean GDT_TS scores of the last ten entries with e-values > 0.1 in the table. For these most difficult targets, SmotifTF has a mean GDT_TS score of 35.24, which is the best along with I-tasser (35.28). If we consider only the entries with e-values > 2.0 (bottom 4 rows), the difference in performance is even more striking with SmotifTF and I-tasser showing the best performance amongst all the methods with an average GDT_TS of 27.65 and 27.77, respectively. As expected, the performance of HHpred drops the most (Mean GDT_TS drops from 31.56 to 19.00), as this method is explicitly dependent on finding a reasonable overall template, while all other methods are able to combine fragments from a larger variety of possible hits. Overall, there seems to exist a trend, which shows that SmotifTF has a better performance compared to the other methods when the difficulty of prediction is greater as expressed by the e-value of the best template available.

**Table 2:** Performance of SmotifTF on the benchmarking test set in comparison to other methods

PDB	N_res_ ^1^	SS^2^	e-value^3^	SmotifTF^4^	I-tasser^4^	HHpred^4^	Rosetta^4^
4v1am	109	Mainly-α	0.000001	47.64	53.21	55.28	30.73
4rd5A	156	α+β	0.006	16.67	17.31	20.35	20.99
2mpvA	145	Mainly-β	0.0098	25.71	55.69	38.10	12.93
4ux3B	64	α+β	0.029	25.00	32.81	42.58	32.42
4nknA	116	Mainly-α	0.039	34.55	22.61	20.00	50.00
3wzsA	140	α+β	0.072	26.79	57.14	47.68	29.46
4wwrA	49	Mainly-α	0.12	57.65	52.55	54.08	49.49
4pqzA	131	Mainly-α	0.17	34.92	23.47	20.61	27.10
4ro3A	103	α+β	0.27	41.26	55.58	53.88	34.95
4uzxA	54	Mainly-α	0.41	56.94	61.57	37.96	52.78
4waiA	82	Mainly-α	0.79	30.18	35.67	26.52	33.84
4o7kA	190	α+β	0.8	20.83	12.90	11.97	19.34
2mpoA	182	Mainly-β	2.1	22.35	15.52	10.30	12.50
4ndsA	74	α+β	2.4	32.43	43.24	29.05	33.78
4qtnA	236	Mainly-α	2.6	21.78	22.69	15.42	18.51
4wyqA	119	Mainly-α	5.5	34.03	29.62	21.22	32.35
Mean (all rows)							
	122.50		0.96	33.05	36.97	31.56	30.70
Mean (rows with e-value of best hit > 0.1)							
	132.00		1.52	35.24	35.28	28.10	31.46
Mean (rows with e-value of best hit > 2.0)							
	152.75		3.15	27.65	27.77	19.00	24.29

^1^ = Number of residues in the query protein

^2^ = Major secondary structure class according to DSSP [57]

^3^ = e-value of the best hit in the dynamic database

^4^ = GDT_TS score of the best scoring model when compared to the native structure.

While the amount of data is not sufficient to draw statistically conclusive results, nevertheless, from among the 10 the most difficult targets (with e-values to the best PDB hit above 0.1), SmotifTF has the most accurate models amongst the methods compared in four out of five large targets (sizes 119, 131, 182, 190, 236 in Table 2), and in the fifth case, it is a close second.

We calculated the relative contact order [56] for the target proteins in Table 2 but no apparent correlation could be seen when comparing it with accuracy. We also identified the protein classes for these targets as shown in Table 2. Among the 16 proteins there are 8, 2 and 6 cases that are mainly-α, α+β and mainly-β classes, respectively.

In terms of the time scales of the four different methods, HHpred server is the fastest, providing results within the order of minutes for all proteins in our benchmark set. I-tasser server, due to its intensive public use and waiting period, provided results within 24–48 hours after submission. SmotifTF and Rosetta were carried out using our in-house linux cluster with 100 computing cores. While SmotifTF completed all jobs within 6–12 hours, Rosetta completed most jobs within 12–24 hours.

### Details of individual modeling cases

#### (a) N-terminal domain of a protein with unknown function from *Vibrio Cholerae* (PDB: 4ro3A)

**Fig 3:**
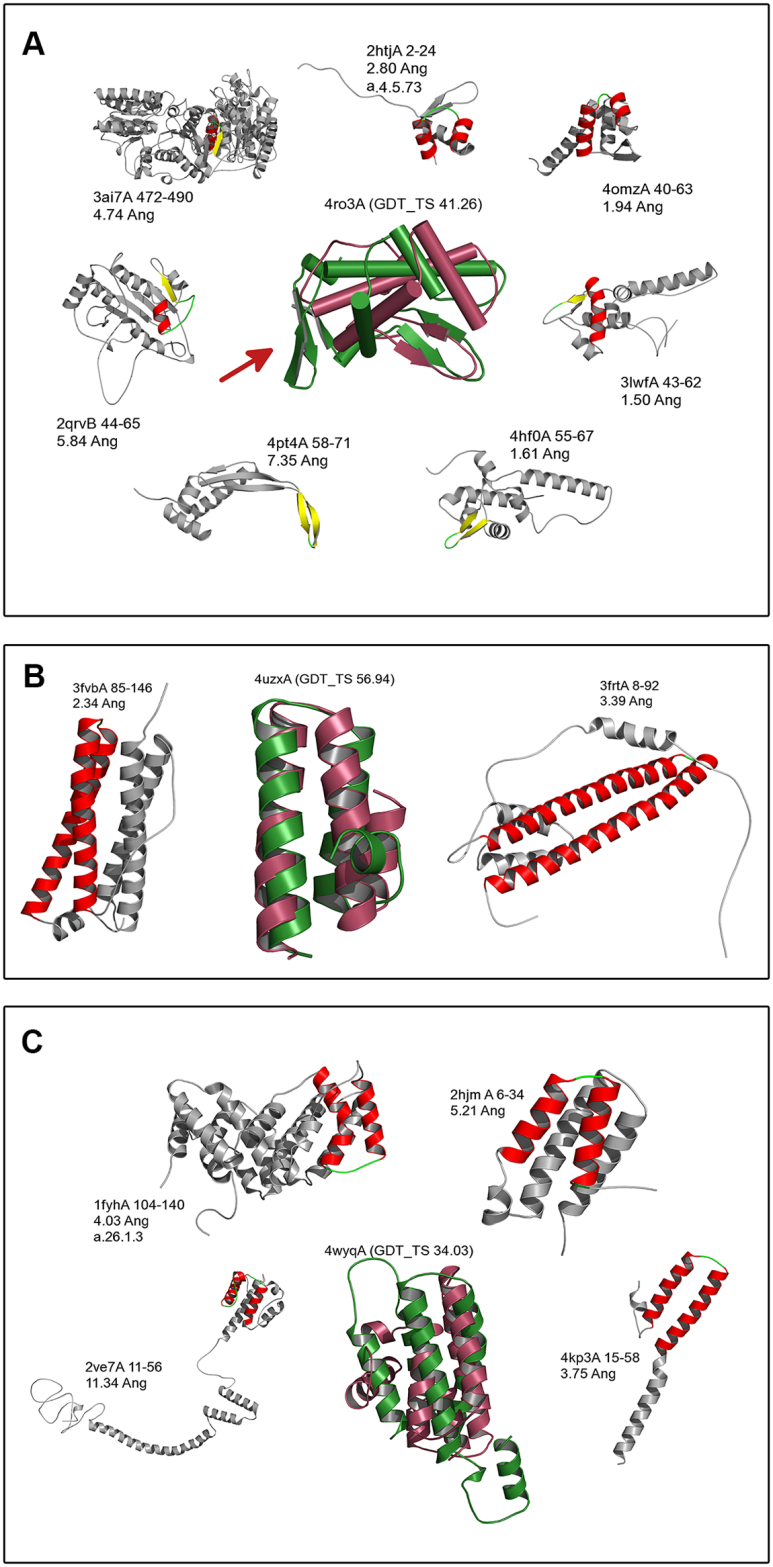
Examples of SmotifTF predictions in the benchmark test set. The structural superposition of the top-scoring model (pink cartoon) with the native structure (green cartoon) is shown in the middle. The proteins that provide the Smotif fragments to the top-scoring model are shown in grey cartoon, with the Smotif themselves colored according to the secondary structure elements present in them (helix = red, strand = yellow, loop = green). The PDB id, chain id and residue numbers of the Smotif fragments are shown along with the root mean square deviation (RMSD) of the respective Smotif fragments compared to the corresponding native Smotif. The SCOP ids of the proteins are provided, where available. (a) N-terminal domain of a protein with unknown function from *Vibrio Cholerae* (PDB: 4ro3A) (b) RNA binding protein Tho1 from *Saccharomyces Cerevisiae* (PDB: 4uzxA) (c) Mammalian Endoribonuclease Dicer (PDB: 4wyqA).

This is an α+β protein with 103 residues. It has 7 Smotifs as identified from PSIPRED. The best prediction from SmotifTF has a GDT_TS of 41.26 and is better than the Rosetta prediction although I-tasser and HHpred have even better models (Table 2). Most of the protein core, including a beta hairpin, has been captured correctly in the model as shown in the structural superposition with the native in Fig 3a. The errors mainly occur in the small beta sheet region (marked by a red arrow), which has been incorrectly assigned in the PSIPRED prediction. Fig 3a also shows the structures of the proteins that contributed fragments to the assembly of this model. The Smotif fragments are found in very different protein structures, which are also different from the structure of the query protein itself. This illustrates the hypothesis that the existing Smotif fragments can be used to model new and existing protein folds.

#### (b) RNA binding protein, Tho1, from *Saccharomyces Cerevisiae* (PDB: 4uzxA)

This is a small α-helical protein with 54 residues and two Smotifs. Here, the SmotifTF prediction produced a very accurate model (GDT_TS score: 56.94; Fig 3b). Except for the small helix at the C-terminus, the rest of the structure is captured correctly in the model. I-tasser predicts a better model with GDT_TS score of 61.57 (Table 2).

#### (c) Mammalian Endoribonuclease dicer (PDB: 4wyqA)

This is one of the toughest target in the benchmarking dataset where the e-value of the best hit is 5.5, indicating that the Smotif fragments come from protein structures that are evolutionarily distant or unrelated to the target protein. SmotifTF gives the best prediction with a GDT_TS of 34.03 (Fig 3c), with Rosetta, I-tasser and HHpred following with 32.35, 29.62 and 21.22, respectively. The structure superposition shows that while SmotifTF is able to predict the some of the long helices in the core correctly, the terminal helices are incorrectly captured. This example shows that as the difficulty of the targets increase, SmotifTF fragment assembly is able to step up and provide better predictions compared to other methods.

If we examine the Smotif fragments that make up the best scoring model and the proteins that contributed them to the dynamic database pool, we find that out of the 99 query Smotifs in the benchmark data set (belonging to 16 test proteins), 66 best scoring Smotifs have been identified from HHblits/HHsearch, 27 have been identified using delta-BLAST and 15 have been identified using psi-BLAST (the total adds up to more than 100 because some hits are identified by more than one method). Although the SmotifTF prediction algorithm starts with the fragments provided by HHblits/HHsearch, the final predictions have improved upon the HHpred predictions in many cases, where the same HHblits/HHsearch results are used as homology modeling templates. This improvement is likely due to the fact that unlike HHpred, SmotifTF follows a fragment assembly method and the Smotif fragments themselves are sampled from many different proteins identified using HHblits/HHsearch, Psi-BLAST and delta-BLAST.

### Factors affecting model quality

#### (a) Secondary structure prediction method

The SmotifTF prediction algorithm depends heavily on the quality of secondary structure prediction method, PSIPRED [43], to identify the putative Smotifs in the query protein. Accurate definition of Smotifs is essential to the method and even minor errors in this first step can lead to drastic differences in the final predictions (example: 4ro3, Fig 3a, Table 2). This is compounded by the fact that, most secondary structure prediction methods themselves rely upon the existence of homologous sequences in the structure database and hence they falter more frequently in case of *ab initio* or template-free modeling targets, where reliable homologs cannot be found.

#### (b) Size of the protein

It has been observed earlier that template-free prediction methods perform well when the protein size is below 120 amino acids [10, 18]. In a similar trend, the SmotifTF method is also able perform better for proteins of smaller size, with the quality of prediction decreasing with larger proteins (Table 2). However, one major difference is that the SmotifTF predictions are better for small proteins with less number of Smotifs because effective sampling can be achieved in these cases even with the limited number of fragments in the Smotif library. For instance, 4wwr and 4uzx are two proteins in our test set where SmotifTF has predicted models with GDT_TS above 50.00 and both are small proteins with just two Smotifs each (Table 2). With large proteins, where sampling is computationally more expensive, the SmotifTF prediction algorithm provides models of lower quality, although in some cases, it is the best performing method amongst the four different methods compared (4o7k, 2mpo in Table 2). Another important factor specific to the SmotifTF prediction algorithm is that sampling is affected mainly by the number of Smotifs in the query protein rather than the actual size of the protein in terms or the number of residues. This sometimes helps us in better predictions for large proteins that have a more regular structure with fewer Smotifs. For instance, 4pqz (Table 2) has 131 amino acids but only 3 Smotifs and SmotifTF has a relatively good prediction for this protein with a 34.92 GDT_TS model.

#### (c) Smotif ranking using HHalign

The SmotifTF prediction algorithm uses HHblits [46] and HHalign [45] to obtain and align Hidden Markov Model (HMM) profiles of the query Smotifs to the Smotifs in the dynamic library. The method further relies on the e-values provided by HHalign to rank the Smotif fragments in the library, which is then used to choose the best fragments for the decoy sampling step. The e-values provided by HHalign fail to pick the best available fragment in the library (in terms of RMSD to the query Smotif) in 66 of the 99 Smotifs in the benchmarking data set. However, for 43 of these 66 Smotifs, the method does sample a library Smotif within 1Å of the best available one, thereby neutralizing the effect of the missed fragment.

## Discussion

New methods for template-free modeling are needed to advance computational techniques of protein structure predictions. We have developed a novel method that uses a fragment library of supersecondary structure motifs to model protein structures. The method follows a fragment assembly protocol using a tailor-made library of supersecondary structure fragments obtained from remotely related proteins using weak sequence signals. The method predicts the core of the protein and the overall fold correctly in over 75% of the cases in the training set and in 50% of the cases explored in the benchmark test set of template-free targets. We have also shown that the current method performs competitively when compared to other existing methods of template-free prediction, which were the best performers in recent CASP experiments. Further, as the difficulty of prediction increases, the Smotif-based template-free prediction method performs better than the other methods compared. The method is relatively simple compared to some other existing approaches, and its good performance is mainly acknowledged to the idea of using an exhaustive set of supersecondary structure fragments. The Smotif-based prediction algorithm is a promising approach to address one of the most challenging problems in molecular biology.

## Materials and Methods

### Smotif system representation

The foundation of the SmotifTF method lies in the representation of protein structures as a set of overlapping supersecondary structure motifs or Smotifs. Smotifs are defined as two regular secondary structure elements (helices and strands) in a protein connected by a loop. In a previous study [37], we had built a library of Smotifs from all known protein structures in the Protein Data Bank [5]. This library consists of over 500,000 individual Smotifs classified based on the type of the bracing secondary structure elements (HH, HE, EH and EE) and grouped into a few thousand clusters based on their internal geometry. The Smotif library is a backbone-only, geometrically defined fragment library with no side-chain information.

### Prediction algorithm

The overall prediction method is summarized in Fig 1. The main aspects of the current method are: (a) A dynamic library of Smotifs is built for each query protein using weak sequence signals to remote homologs. (b) The weak sequence signals are further used to identify suitable fragments from the dynamic Smotif library for sampling (c) Sampling of full protein conformations is explored using exhaustive enumeration of all possible combinations of the fragments chosen earlier (d) The sampled full protein structures are scored using a composite energy function to identify the best scoring model.

#### (a) Building the dynamic Smotif library

A “dynamic” library of Smotif fragments is built specifically for each query protein, from all known PDB structures. The dynamic library is built using sequence information from three sequence-based methods run with default parameters: Psi-BLAST [31], delta-BLAST [44], HHblits/HHsearch [45, 46]. All three methods provide confidence measures in terms of e-values, which can be interpreted as the likelihood that the observed result occurred by chance. The templates provided by the three methods are pooled together and the proteins are further broken down into their constituent Smotif fragments. These fragments constitute the dynamic library of Smotifs, corresponding to the particular query protein (Fig 1). Missing loops in PDB files (which occur often) can lead to the loss of Smotif fragments in the library. To overcome this issue, all the missing loops in the entire PDB are built using Modeller [55].

#### (b) Identifying Smotif fragments from the dynamic database

The secondary structures in the query protein are predicted using PSIPRED [43] and the putative Smotifs in the query protein are identified (Fig 1). Next, we search for suitable Smotif fragments from the dynamic library for each Smotif in the query protein using Hidden Markov Model (HMM) profiles built from HHblits search [46]. For every query Smotif, a set of Smotifs from the dynamic library is selected that (i) belongs to the same Smotif type as the query Smotif (ii) the loop lengths of the query and the library Smotifs match with a +/- 1 deviation. The HMM profiles of the shortlisted library Smotifs are aligned to that of the query Smotif using HHalign [45]. HHalign provides a reliability score in terms of the e-value, which is used to the rank the library Smotifs. The top 4–24 Smotifs are selected for each query Smotif based on the e-value ranking (Fig 1). The number of Smotifs selected for model building is varied depending on the size of the query protein to generate about a million models in total. For smaller proteins (with fewer than six query Smotifs), a larger set of fragments (around 12–24 per Smotif) is selected and for larger proteins (with more than six query Smotifs), a smaller set of fragments is selected (around 4–8 per Smotif).

#### (c) Sampling and scoring of Smotif combinations

Since the Smotif fragments are large in size (average Smotif size is 27.44 amino acid residues in the test set used), it allows us to carry out an exhaustive enumeration of all possible combinations of the Smotif fragments chosen in the previous step. Successive Smotifs are joined by optimally superposing their overlapping secondary structures. Length of secondary structures of the sampled Smotifs are extended or shortened to fit the query sequence. In the process of joining Smotifs, a limited number of steric clashes (equal to the number of total Smotifs in the structure) are allowed.

(c) In the next step, a composite scoring function is used to score and rank the million sampled models to identify the best model. The scoring function consisting of a linear combination of four different components: radius of gyration using Cα carbons, an orientation dependent statistical potential [47–49], a knowledge-based long-range backbone hydrogen bond potential [50] and an implicit solvation potential [51]. All components are converted into statistical Z-scores before combining them. The weights for the linear scoring function were optimized on a set of decoy structures obtained from five proteins of varying sizes and secondary structure composition (1PTF, 1M7T, 1ZLM, 2LIS, and 2DC3), all of which were disjoint from the proteins used to develop this algorithm. The best scoring structures from this scoring scheme are relaxed using Modeller [55] to resolve steric clashes, fix side-chains and maintain stereochemistry.

### Benchmarking the algorithm

We developed the algorithm on a set of 20 proteins (Table 1). These were randomly chosen to represent proteins from different folds. *Ab initio* conditions were simulated by systematically removing high-quality templates from the dynamic Smotif library with e-values better than 10^-10^, 10^-5^, 10^-1^ and 10^0^, respectively (Table 1).

The method was further tested on a new set of 16 proteins (Table 2). These were specifically chosen to be *ab initio* targets from new PDB structures released each week starting from 10-8-2014 to 12-31-2014. The sequences of new PDB releases were obtained each week, clustered using CD-hit [58] at 90% to eliminate similar sequences and then tested using psi-BLAST [31] and HHsearch [45] against the rest of the PDB to eliminate homology modeling targets. The predictions of the SmotifTF algorithm were compared to three other prediction methods: I-tasser [12], Rosetta [11] and HHpred [52], which performed well in recent Critical Assessment of Structure Prediction (CASP) experiments [18, 29]. We have chosen new weekly releases from the PDB and focused on those proteins with no templates in the PDB (other than itself) to identify *ab initio* targets. The advantage of doing this is that it mimics blind testing of the methods with minimal intervention from already existing templates in the PDB.

### Software availability

SmotifTF is a free software package created using Perl and is distributed under the Artistic license version 2.0 (GPL compatible). The complete package can be downloaded from the Comprehensive Perl Archive Network at http://search.cpan.org/dist/SmotifTF/. The current version supports multiple cores for parallel computing.
